# Identification of KLF9 and BCL3 as transcription factors that enhance reprogramming of primordial germ cells

**DOI:** 10.1371/journal.pone.0205004

**Published:** 2018-10-04

**Authors:** Kei Otsuka, Asuka Takehara, Natsuko Chiba, Yasuhisa Matsui

**Affiliations:** 1 Cell Resource Center for Biomedical Research, Institute of Development, Aging and Cancer (IDAC), Tohoku University, Sendai, Miyagi, Japan; 2 Department of Cancer Biology, Institute of Development, Aging and Cancer, Tohoku University, Sendai, Miyagi, Japan; 3 The Japan Agency for Medical Research and Development-Core Research for Evolutional Science and Technology (AMED-CREST), Chuo-ku, Tokyo, Japan; 4 Graduate School of Life Sciences, Tohoku University, Sendai, Miyagi, Japan; 5 Center for Regulatory Epigenome and Diseases, Tohoku University School of Medicine, Sendai, Miyagi, Japan; Macau University of Science and Technology, MACAO

## Abstract

Primordial germ cells (PGCs) are precursors of eggs and sperm. Although PGCs are unipotent cells *in vivo*, they are reprogrammed into pluripotent stem cells (PSCs), also known as embryonic germ cells (EGCs), in the presence of leukemia inhibitory factor and basic fibroblast growth factor (bFGF) *in vitro*. However, the molecular mechanisms responsible for their reprogramming are not fully understood. Here we show identification of transcription factors that mediate PGC reprogramming. We selected genes encoding transcription factors or epigenetic regulatory factors whose expression was significantly different between PGCs and PSCs with in silico analysis and RT-qPCR. Among the candidate genes, over-expression (OE) of *Bcl3* or *Klf9* significantly enhanced PGC reprogramming. Notably, EGC formation was stimulated by *Klf9*-OE even without bFGF. G-protein-coupled receptor signaling-related pathways, which are involved in PGC reprogramming, were enriched among genes down-regulated by *Klf9*-OE, and forskolin which activate adenylate cyclase, rescued repressed EGC formation by knock-down of *Klf9*, suggesting a molecular linkage between KLF9 and such signaling.

## Introduction

Cellular reprogramming to establish pluripotent stem cells (PSCs) is a topic in basic research and may have medical applications. Various types of differentiated somatic cells can be reprogrammed into induced pluripotent stem cells (iPSCs) by forced expression of pluripotency-associated genes such as *Oct4*, *Klf4*, *Sox2*, and *cMyc*, which are known as the Yamanaka factors [1,2]. For several decades prior to identification of the Yamanaka factors, classical genetic studies in mice suggested that germ cells in mouse embryos can be converted into PSCs that develop into a teratoma, which consists of various tissues and cells that differentiated from PSCs. Teratomas develop in the gonads of mice on specific genetic backgrounds such as the 129/Sv strain and/or by mutations of genes including *Dead-end 1* (*Dnd1*) [3,4]. More recently, analysis of *Dnd1* functions has revealed the molecular mechanisms of conversion of germ cells into pluripotent early teratoma cells in embryos, including control of genes involved in enhancement of the cell cycle in germ cells [5,6]. In addition, reprogramming of germ cells into PSCs is recapitulated in culture when primordial germ cells (PGCs) or spermatogonial stem cells are cultured with specific cytokines [7–9], indicating that extra-cellular stimuli are sufficient to induce reprogramming of germ cells.

In initial studies, reprogramming of mouse PGCs to pluripotent embryonic germ cells (EGCs) was induced by addition of leukemia inhibitory factor (LIF) and basic fibroblast growth factor (bFGF) with serum on feeder cells expressing membrane-bound Steel factor also known as stem cell factor and kit ligand. Subsequent studies revealed that bFGF can be replaced by retinoic acid (RA) or forskolin (FK: an activator of adenylate cyclase) [10], Trichostatin A (an inhibitor of histone deacetylase) [11], inhibitors of mitogen-activated protein kinase signaling and an inhibitor of glycogen synthase kinase 3 (2i) [12], or an inhibitor of transforming growth factor beta receptor [13]. EGCs can also be established in the presence of 2i, LIF, bFGF, stem cell factor, RA, and FK on fibronectin-coated culture dishes without serum and feeder cells [14]. In addition, activation of Akt, a critical intra-cellular signaling molecule, enhances the efficiency of EGC formation from PGCs and partially replaces LIF or bFGF [15,16]. The results together indicate that a specific intra-cellular signaling status is crucial for reprogramming of PGCs, although the detailed molecular mechanisms are not fully understood.

In addition to intra-cellular signaling, the importance of transcriptional regulation including repression of the transcription factor, BLIMP1 playing a role on PGC specification [17], for PGC reprogramming has also been suggested. *Blimp1* is down-regulated shortly after PGC reprogramming begins [11]. In addition, deletion of *Blimp1* enhances PGC reprogramming, and BLIMP1 represses the pluripotency network in embryonic stem cells (ESCs), together suggesting that BLIMP1 functions as a pluripotency gatekeeper in PGCs [18]. However, additional transcriptional regulation involved in PGC reprogramming is currently unclear. To address this issue, we searched for transcription or epigenetic regulatory factors that are crucial for PGC reprogramming and found that KLF9 and BCL3 play a role in PGC reprogramming.

## Materials and methods

### Animals

MCH and C57BL/6 mice were purchased from CLEA Japan and Japan SLC, respectively. The Oct4-deltaPE-GFP [19] transgenic mice were maintained in a C57BL/6J genetic background. For collecting PGCs, female MCH mice were mated with male Oct4-deltaPE-GFP mice. Noon on the day of the plug was defined as E0.5. The mice were kept and bred in an environmentally controlled and specific pathogen-free facility, the Animal Unit of the Institute of Development, Aging and Cancer (Tohoku University), according to the guidelines for experimental animals defined by the facility. Animal protocols were reviewed and approved by the Tohoku University Animal Studies Committee.

### Flow cytometry

E12.5 or E13.5 Oct4-deltaPE-GFP transgenic embryos were collected and dissected in Dulbecco’s modified Eagle medium (DMEM, Gibco) containing 10% fetal bovine serum (FBS). The genital ridges of male and female embryos were dissected. Tissue samples containing PGCs, were incubated with 1.2 mg/ml collagenase (SIGMA) in PBS containing 10% FBS for 1h at 37°C. To prepare single-cell suspensions for flow cytometry, tissues were dissociated by pipetting, and were filtered through a nylon mesh (40 μm pore size, BD falcon). A Bio-Rad S3e cell sorter was used to sort and collect PGCs with intense GFP expression.

### ESC culture

Vasa-RFP (VR15) ESCs [20] were cultured in KnockOut DMEM (Gibco) supplemented with 15% FBS, 4 mM L-glutamine (Gibco), 0.01 mM nonessential amino acids (Gibco), 0.1 mM β-mercaptoethanol (SIGMA), 1,000 U/ml LIF (ESGRO Millipore) on mouse embryonic fibroblasts inactivated with mitomycin C (SIGMA). Blimp1-mVenus-Stella-ECFP (BVSC) ESC [21], which were kindly provided from Dr. Mitinori Saitou, were cultured in 2i (PD0325901, CHIR99021) + LIF, feeder-free culture conditions [22].

### RNA preparation and reverse transcription real-time PCR

Total RNA samples were purified using RNeasy Micro Kit (QIAGEN) according to the manufacturer’s instruction. RNAs were reverse-transcribed using SuperScript III (Invitrogen) and random primers (Promega). Expression levels of genes were quantified using the SYBR Green Master Mix (Applied Biosystems) with the primers shown in S1 Table. PCR signals were detected using CFX Connect (Bio-Rad). Transcript levels were normalized relative to those of Arbp.

### Transcriptome analysis

RNA-seq libraries were prepared from 500 ng of total RNA purified from *Klf9*-OE E13.5 PGCs and control PGCs cultured for 1 days, with TruSeq RNA sample prep kit v2 (Illumina). The libraries were clonally amplified on a flow cell and sequenced on HiSeq2500 (HiSeq Control Software v2.2.58, Illumina) with 51-mer single-end sequence. Image analysis and base calling were performed using Real-Time Analysis Software (v1.18.64, Illumina). For gene expression analysis, reads were mapped to the mouse genome (UCSC mm10 genome assembly and NCBI RefSeq database) using TopHat2 and Bowtie. Cufflinks was used to estimate gene expression levels based on reads per kilobase of exon per million mapped reads (RPKM) normalization. Differentially expressed genes (DEGs) were extracted from the Cuffdiff results. The PANTHER (Protein ANalysis THrough Evolutionary Relationships) Classification System ver13.0 (http://pantherdb.org/) was used for pathway analysis, and Venny 2.1 (http://bioinfogp.cnb.csic.es/tools/venny/) was used for Ven diagrams. The microarray data of PGCs, ESCs and 1-day cultured PGCs in previous studies; GEO:assession: GSE30056 (E9.5 PGC and ESC) [22], GSE67616 (E11.5 PGC, 1-day cultured PGCs and ESC) [18], GSE45181 (E13.5 PGC and ESC) [23], were analyzed by using GeneSpring (Agilent).

### Vector construction and production of lentivirus particles

For over-expression (OE) vectors, coding regions of *Bcl3*, *Klf9*, *Nupr1*, *Psrc1*, *Tbx3*, and *Tead4* were amplified from VR15 ESC cDNA using the primer sets shown in S1 Table and sub-cloned into *EcoR* I/*Not* I site of CSII-EF-MCS lentivirus vector by using In-Fusion HD Cloning Kit (Takara Bio) according to the manufacturer’s instructions. For *Bnc1*-, *Isl2-*, *p53-*, and *Klf9-*knock-down (KD) vectors, pairs of oligonucleotides shown in S1 Table were annealed and sub-cloned into *Age* I/ *EcoR* I site of pLKO.1 lentivirus vector. Lentivirus particles were produced as described previously [24]. Briefly, CSII-EF- or pLKO.1- lentivirus vector, pCMV-VSV-G-RSV-Rev and pCAG-HIVgp were co-transfected into HEK293T cells by the calcium phosphate method. For titration of lentiviruses, a Lenti-X qRT-PCR Titration Kit (Takara Bio) was used according to the manufacturer’s instructions. Virus particles were collected by centrifuging the cultured medium at 2,330 × g for 30 minutes at 4°C after incubating with PEG6000 solution [final 2.5% PEG6000 (Wako), 100 mM NaCl, 10 mM HEPES (pH 7.4)] overnight at 4°C, and they were re-suspended in EG medium [StemPro34 SFM (Gibco) containing StemPro34 Nutrient, 100 μg/ml transferrin (SIGMA), 2 mM L-glutamine, 25 μg/ml insulin (SIGMA), 50 μM β-mercaptoethanol, 20 ng/ml EGF (SIGMA), 10% knockout serum replacement (KSR, Gibco), 100 U/ml penicillin-streptomycin (SIGMA), 25 ng/ml human bFGF (SIGMA), and 1,000 U/ml LIF] [16] and stored at -80°C until they were used. In some experiments, they were re-suspended in EG medium without LIF or bFGF. CSII-EF-MCS, CSII-EF-mcherry, or pLKO.1-empty vectors were used as control.

### PGC culture

PGC culture for reprogramming was carried out as described previously [16,25] with some modifications. The sorted E12.5 PGCs were cultured on a feeder layer of Sl/Sl4-m220 cells [25] inactivated with mitomycin C in 24-well tissue culture dishes with EG medium (see above). After 6–7 days in culture, EGC colonies were identified by staining for alkaline phosphatase activity as described previously [16,25]. The efficiency of EGC formation was determined as ratios of EGC colony number in every 100 seeded PGCs in a culture well. Infection of the lentivirus vectors to PGC were carried out as described previously [26] with some modifications. After seeding PGCs with lentivirus, 24-well tissue culture dishes were centrifuged at 1,650 × g for 1 h at 30°C. The multiplicities of infection (MOI) were adjusted to 5 or 0.2. For real-time qPCR and RNA-seq, sorted PGCs at E12.5 and E13.5, respectively were cultured on gelatin-coated 24 well-plates for 1 or 2 days. In some experiments, PGC were cultured with EG medium containing 10μM forskolin (SIGMA).

### Statistical analysis

Statistical analysis was performed using the Student’s t-test. P values < 0.05 were considered to be statistically significant.

## Results

### Candidate genes that regulate PGC reprogramming into EGCs

**Fig 1:**
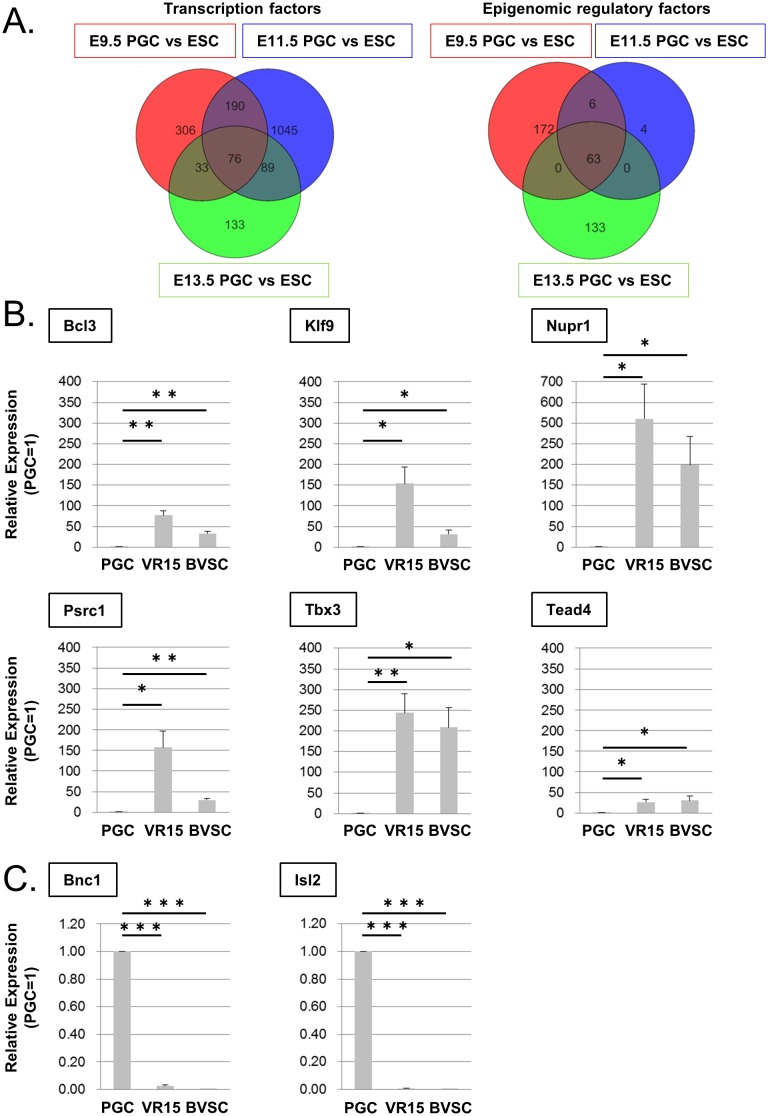
Candidate genes selected by their differential expression in ESCs and PGCs. (A) Venn diagram showing transcription factor genes (left) and epigenetic regulatory factor genes whose expression is different between ESCs and PGCs at E9.5 (red circle), E11.5 (blue circle), and E13.5 (green circle). GEO:assession: GSE30056 (E9.5 PGC and ESC)[22], GSE67616 (E11.5 PGC and ESC)[18], GSE45181 (E13.5 PGC and ESC)[23]. (B, C) Confirmation of differential expression of candidate genes by RT-qPCR. Relative expression levels in VR15-ESCs and BVSC-ESCs compared with those in E12.5 PGCs. Genes whose expression is more than 10 times higher (B) or lower (C) in ESCs than in PGCs are shown. The expression of the remaining genes is shown in S1 Fig. Error bars show the SE of three independent experiments. *p < 0.05, **p<0.01, ***p < 0.001.

To identify candidate genes that control PGC reprogramming into EGCs, we re-analyzed published microarray data [18,22,23] and selected genes that encode transcription factors or epigenetic regulatory factors and whose expression is different between ESCs and PGCs at E9.5, 11.5, and 13.5 from which EGCs can be established. Following this analysis, we selected 25 genes that encode transcription factors (76 probes) and 14 genes that encode epigenetic regulatory factors (63 probes) (Fig 1A and S2 Table).

Next, we confirmed whether the expression of the 39 candidate genes was significantly different between ESCs and PGCs with RT-qPCR. We selected six and two genes whose expression was more than 10 times higher or lower, respectively, in PGCs from E12.5 embryos than in two different ESCs (Vasa-RFP: VR15, Blimp1-mVenus-Stella-ECFP: BVSC) as possibly important genes for PGC reprogramming (Fig 1B and 1C, and S1 Fig).

### Functional evaluation of the candidate PGC reprogramming factor genes

**Fig 2:**
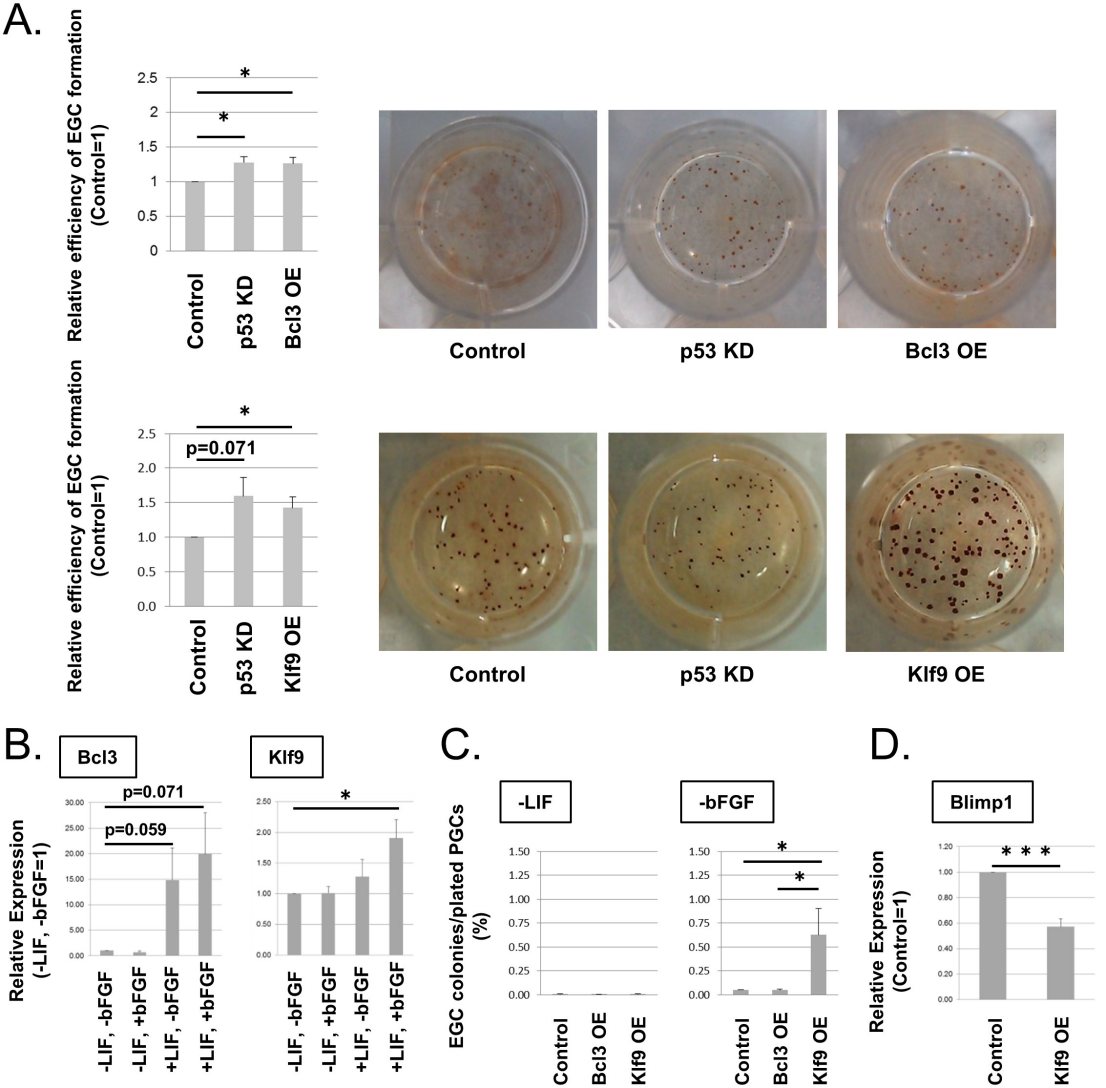
Enhancement of EGC formation by over-expression of *Bcl3* and *Klf9*. (A) Relative efficiency of EGC formation by *Bcl3-*OE or *Klf9-*OE (MOI 5) in PGCs compared with that of control (left). EGC colonies are identified by alkaline phosphatase staining. *p53-*KD is a positive control. The efficiency of EGC formation by OE or KD of the remaining candidate genes is shown in S3 Fig. Error bars show the SE of three (top) and four (bottom) independent experiments. Representative pictures of stained EGC colonies are shown (right). (B) The expression of *Bcl3* and *Klf9* in PGCs cultured for 1 day with (+) or without (−) bFGF and/or LIF. Error bars show the SE of three independent experiments. The expression in PGCs cultured without LIF and bFGF was set as 1.0. (C) Efficiency of EGC formation by *Bcl3-*OE or *Klf9-*OE in PGCs in the presence of bFGF alone (−LIF) or LIF alone (−bFGF). Error bars show the SE of two (−LIF) or four (−bFGF) independent experiments. (D) The relative expression of *Blimp1* in *Klf9*-OE PGCs cultured for 1 day with LIF alone compared with control PGCs. Error bars show the SE of four independent experiments. The expression was determined by RT-qPCR. *p < 0.05, ***p < 0.001.

To evaluate roles of the candidate genes in PGC reprogramming, we tested enhancement of EGC formation by over-expression (OE) or knock-down (KD) of the candidate genes whose expression was up- or down-regulated, respectively, in ESCs compared with PGCs. For OE or KD, we infected E12.5 PGCs with lentivirus vectors and cultured the cells in conditions required for EGC formation. *p53*-KD, which enhances PGC reprogramming by suppressing apoptosis [26], was included as a positive control. Among the candidates, we found that *Bcl3*-OE and *Klf9*-OE significantly enhanced EGC formation (Fig 2A). We confirmed their OE with RT-qPCR (S2A Fig). The remaining genes did not show significant enhancement of EGC formation (S3 Fig).

The expression levels of *Bcl3* and *Klf9* in ESCs were 30~150 folds higher than those in PGCs (Fig 1B), but OE resulted in much higher expression of *Bcl3* and *Klf9* (13,000~25,000 folds compared with control; S2A Fig), when we infected the virus vectors at the multiplicities of infection (MOI) 5. We tested lower MOI, and found that MOI 0.2 resulted in 10~25 folds higher expression (S2B Fig). Even in this condition, EGC formation was still significantly enhanced by *Klf9*-OE (S2C Fig). The enhancement of EGC formation by *Bcl3*-OE at MOI 0.2 was not statistically significant (S2C Fig), which may be due to unstable upregulation of Bcl3 at low levels in this condition (S2B Fig).

We also tested possible additional effects of *Bcl3*-OE and *Klf9*-OE, but concomitant OE of those two genes did not significantly enhance EGC formation compared with *Bcl3*-OE or *Klf9*-OE alone (S2D Fig). It suggest that BCL3 and KLF9 share common downstream pathways.

To examine whether LIF and bFGF, which are essential cytokines for EGC formation, induce the expression of *Bcl3* and *Klf9*, we tested the expression of *Bcl3* or *Klf9* in E12.5 PGCs cultured with or without LIF and bFGF for 1 day. *Bcl3* was highly up-regulated by LIF alone, but bFGF showed no obvious effect on *Bcl3* expression. *Klf9* was up-regulated only when both LIF and bFGF were added (Fig 2B). The results suggest that *Bcl3* is up-regulated in response to LIF signaling, whereas up-regulation of *Klf9* requires both LIF and bFGF signaling.

Next, we examined whether *Klf9*-OE or *Bcl3*-OE can replace bFGF and/or LIF during EGC induction. In the presence of bFGF alone (−LIF in Fig 2C), EGCs were rarely formed with *Bcl3*-OE or *Klf9*-OE. However, *Klf9*-OE but not *Bcl3*-OE increased the efficiency of EGC formation from 0.05 ± 0.01% in the control to 0.62 ± 0.22% with LIF alone (−bFGF in Fig 2C), although the efficiency was lower than in the presence of both LIF and bFGF without OE (routinely about 1.5%). These data suggest that bFGF is involved in multiple signaling pathways for PGC reprogramming. Because previous studies indicated the importance of *Blimp1* down-regulation shortly after PGC reprogramming begins [11,18], we tested whether *Klf9*-OE influenced *Blimp1* expression. *Blimp1* in PGCs was significantly down-regulated after 1 day in culture with *Klf9*-OE (Fig 2D), suggesting that KLF9 is involved in an initial step of PGC reprogramming. Because the enhancement of EGC formation by *Klf9*-OE without bFGF was remarkable compared with that by *Bcl3*-OE with or without LIF and bFGF, we focused on *Klf9* and further examined its possible downstream pathways in this study.

### The cAMP pathway functions downstream of KLF9 during EGC induction

**Fig 3:**
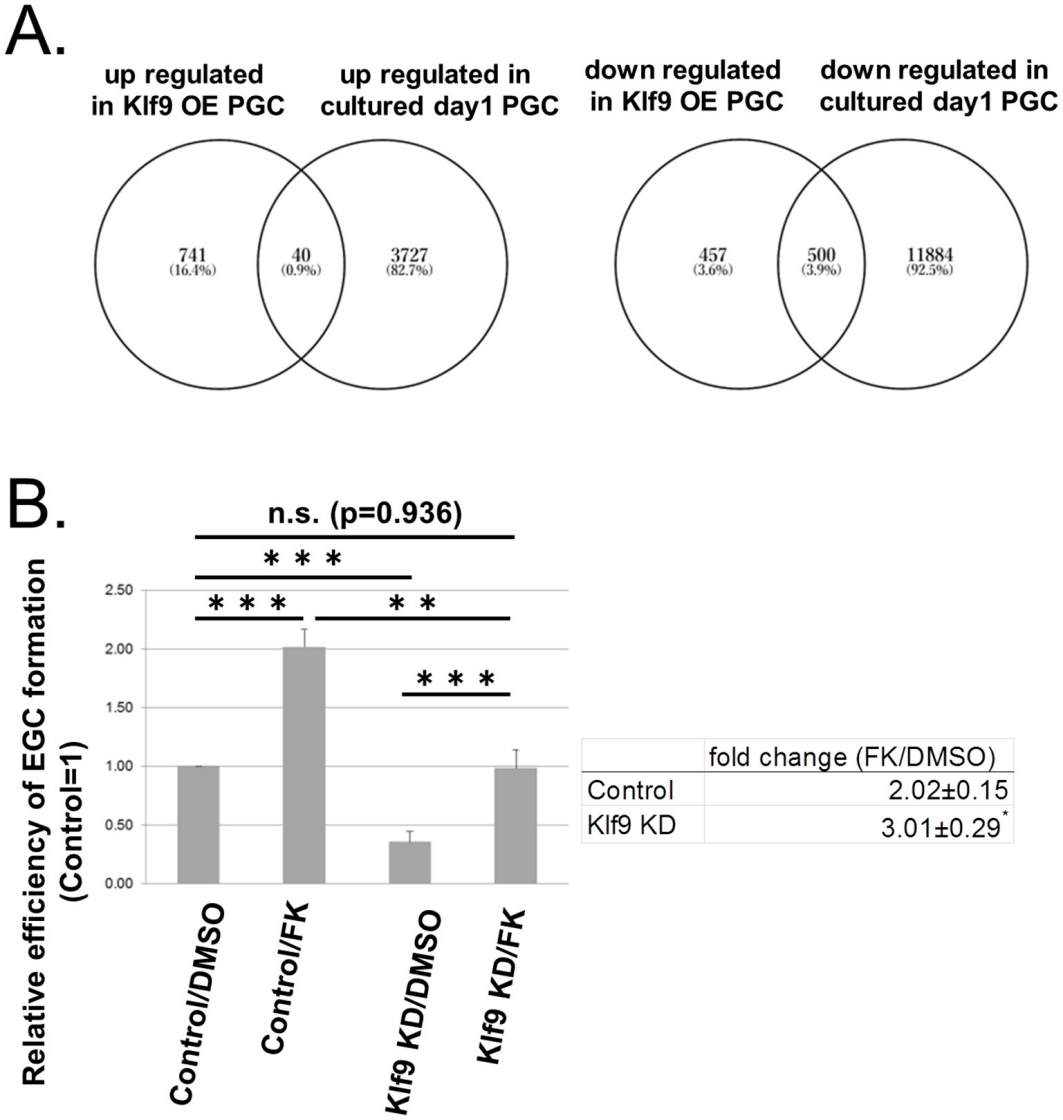
cAMP signaling functions downstream of KLF9. (A) Venn diagrams show the overlap between genes that were up-regulated (left) or down-regulated (right) in *Klf9*-OE PGCs and 1-day cultured PGCs (GSE67616) [18]. (B) The effect of FK on EGC formation from *Klf9*-KD PGCs. Relative efficiency of EGC formation in each condition compared with that in the control culture (Control/DMSO) is shown (left). EGCs were identified by alkaline phosphatase staining. Fold changes in the efficiency of EGC formation by FK with or without *Kfl9*-KD over control (DMSO) are shown (right). Error bars show the SE of five independent experiments. **p < 0.01, ***p < 0.001.

To investigate downstream pathways of KLF9 during EGC induction, we carried out RNA sequencing analysis to select genes that were up- or down-regulated by *Klf9*-OE in E13.5 PGCs cultured for 1 day in the conditions for EGC induction but without bFGF (the DDBJ/GenBank/EMBL; DRA006497). We identified 781 and 959 differentially expressed genes with two times higher or lower expression, respectively, in *Klf9*-OE PGCs compared with control PGCs (S3 and S4 Tables). Because a previous study indicated that the presence of bFGF during the first day in culture is critical for EGC formation [27], and we found that KLF9 replaced bFGF at least to some extent (Fig 2C), we hypothesized that important genes downstream of KLF9 likely changed their expression during the first day of EGC formation. Based on this idea, we analyzed published data (GSE67616) [18], and selected genes that were up- or down-regulated in PGCs after 1 day in culture with LIF and bFGF compared with those before culture. We then extracted genes that changed their expression with *Klf9*-OE and in the 1-day culture (Fig 3A) and further selected candidate genes downstream of KLF9 using pathway analysis. Although pathway enrichment was not found among the commonly up-regulated genes, we found significant enrichment of G-protein-coupled receptor (GPCR) signaling-related pathways such as GPCR ligand binding and G alpha (i) signaling events among genes that were commonly down-regulated by *Klf9*-OE and after 1-day culture (S5 Table).

Considering that bFGF can be replaced by a combination of RA and FK, and FK stimulates cAMP signaling during EGC induction [10], we further examined the possible relationship between KLF9 and GPCR/cAMP signaling and tested the effect of FK with or without *Klf9*-KD in the conditions for EGC induction. As expected, FK alone stimulated and *Klf9*-KD alone repressed EGC formation (Fig 3B, left panel). FK also enhanced EGC formation in the *Klf9*-KD condition, and this enhancement was significantly greater than that without *Klf9*-KD (Fig 3B, right panel). These observations suggest that FK rescues the suppressive effect of *Klf9*-KD during EGC induction, which supports the idea that cAMP signaling may function downstream of KLF9.

## Discussion

In this study, we reported that KLF9 and BCL3, both of which show higher expression in ESCs than in PGCs, enhanced PGC reprogramming to EGCs. BCL3 is a member of the IκB family and interacts with NF-κB to control its functions [28]. In ESCs, *Bcl3* is induced by LIF and plays a role in the maintenance of pluripotency via induction of Oct4 [29], which is consistent with the up-regulation of *Bcl3* by LIF in PGCs (Fig 2B). Because BCL3 stimulates cell-cycle progression in a breast cancer cell line [30,31], and cell-cycle enhancement stimulates EGC formation [5, 6], BCL3 likely enhances PGC reprogramming via cell-cycle control. Even though LIF induces *Bcl3* expression in PGCs, *Bcl3* expression cannot replace LIF for PGCs (Fig 2B and 2C). These observations suggest that additional factors down-stream of LIF other than BCL3 are required for reprogramming of PGCs. More specifically, BCL3 may be unstable only in the presence of either LIF or bFGF. BCL3 is degraded through the proteasome pathway after glycogen synthase kinase 3β-mediated phosphorylation, which is inhibited by Akt [32]. PGC reprogramming is enhanced by Akt, and both LIF and bFGF may be involved in Akt activation. Therefore, not only bFGF but also LIF are likely required for fully activated Akt to prevent BCL3 degradation.

KLF9 is a member of the Krüppel-like factor family of transcription factors. Among KLFs, KLF2, 4, and 5 belong to group 2 of the KLF family and are involved in pluripotency transcription networks in ESCs and iPSCs [1,2,33,34]. KLF9 is a member of group 3 based on its N-terminal structure [34]. The functions of KLF9 in PSCs are unknown. Although *Klf9*-OE enhanced PGC reprogramming in the absence of bFGF, the efficiency of EGC colony formation by *Klf9*-OE without bFGF was lower than that with LIF and bFGF (Fig 2C). In addition, the expression of *Klf9* was not induced by bFGF alone. The results suggest that KLF9 partially replaced bFGF functions for PGC reprogramming, and different signaling molecules other than KLF9 may function downstream of bFGF.

We also found that GPCR signaling-related pathways including a repressive G alpha (i) pathway were enriched among genes commonly down-regulated by *Klf9*-OE and after 1-day culture of PGCs in reprogramming conditions (S5 Table). These data suggest that KLF9 enhances cAMP signaling via repression of G alpha (i)-related genes (highlighted in S4 Table). Previous studies indicated that FK, which increases intra-cellular cAMP, enhances PGC growth and survival as well as their reprogramming [10,35,36]. Our results suggest that FK partially rescues decreased EGC formation by *Klf9*-KD. Therefore, activation of cAMP signaling may be involved in enhancement of EGC formation by *Klf9*-OE. The detailed molecular mechanisms of cAMP-dependent enhancement of PGC reprogramming remain to be determined in future studies.

## Supporting information

S1 Fig The expression of candidate genes in PGCs and ESCs. The expression of candidate genes was determined with RT-qPCR, and relative expression levels in VR15-ESCs and BVSC-ESCs compared with those in E12.5 PGCs are shown. Genes whose expression is less than 10 times higher (A) or lower (B) in ESCs compared to PGCs are shown. Error bars show the SE of three independent experiments. *p<0.05, **p<0.01, ***p<0.001.
(TIF)

S2 Fig The expression levels of *Bcl3* or *Klf9* induced by infection with lentivirus vectors with different MOI, and their influences for EGC formation. (A, B) Induction of *Bcl3* or *Klf9* expression in *Bcl3-*OE or *Klf9-*OE PGCs by infecting the lenti-virus vectors at MOI 5 (A) and MOI 0.2 (B) after culturing for 2 days. The expression was determined by RT-qPCR. (C, D) Relative efficiency of EGC formation by *Bcl3-*OE or *Klf9-*OE (MOI 0.2) (C) or by *Bcl3-*OE and/or *Klf9-*OE (MOI 5) (D) in PGCs compared with that of control. EGC colonies are identified by alkaline phosphatase staining. Error bars show the SE of four (A, C, D), two (B) independent experiment. *p < 0.05, **p < 0.01, ***p < 0.001.
(TIF)

S3 Fig The effect of KD or OE of the candidate genes on EGC formation. Relative efficiency of EGC formation by *Bnc1*-KD, *Isl2*-KD, *Nupr1*-OE, *Psrc1*-OE, *Tead4*-OE, and *Tbx3*-OE PGCs compared with control is shown. Error bars show the SE of three independent experiment. *p < 0.05. **p < 0.01.
(TIF)

S1 Table List of primers used in this study. (XLSX)

S2 Table Transcription factor genes and epigenetic regulatory factor genes whose expression is different between ESCs and PGCs at E9.5, E11.5, and E13.5). (XLSX)

S3 Table A list of up-regulated genes by *Klf9*-OE in PGCs. (XLSX)

S4 Table A list of down-regulated genes by *Klf9*-OE in PGCs. Genes highlighted in gray are G alpha (i)-related genes.
(XLSX)

S5 Table Pathway analysis of genes commonly down-regulated by *Klf9-*OE and 1-day culture of PGCs. (XLSX)
