# Supplementary figures and images for: Identification of KLF9 and BCL3 as transcription factors that enhance reprogramming of primordial germ cells
Source: PLoS One. 2018 Oct 4;13(10):e0205004. doi: 10.1371/journal.pone.0205004 (PMC6171932; doi:10.1371/journal.pone.0205004)

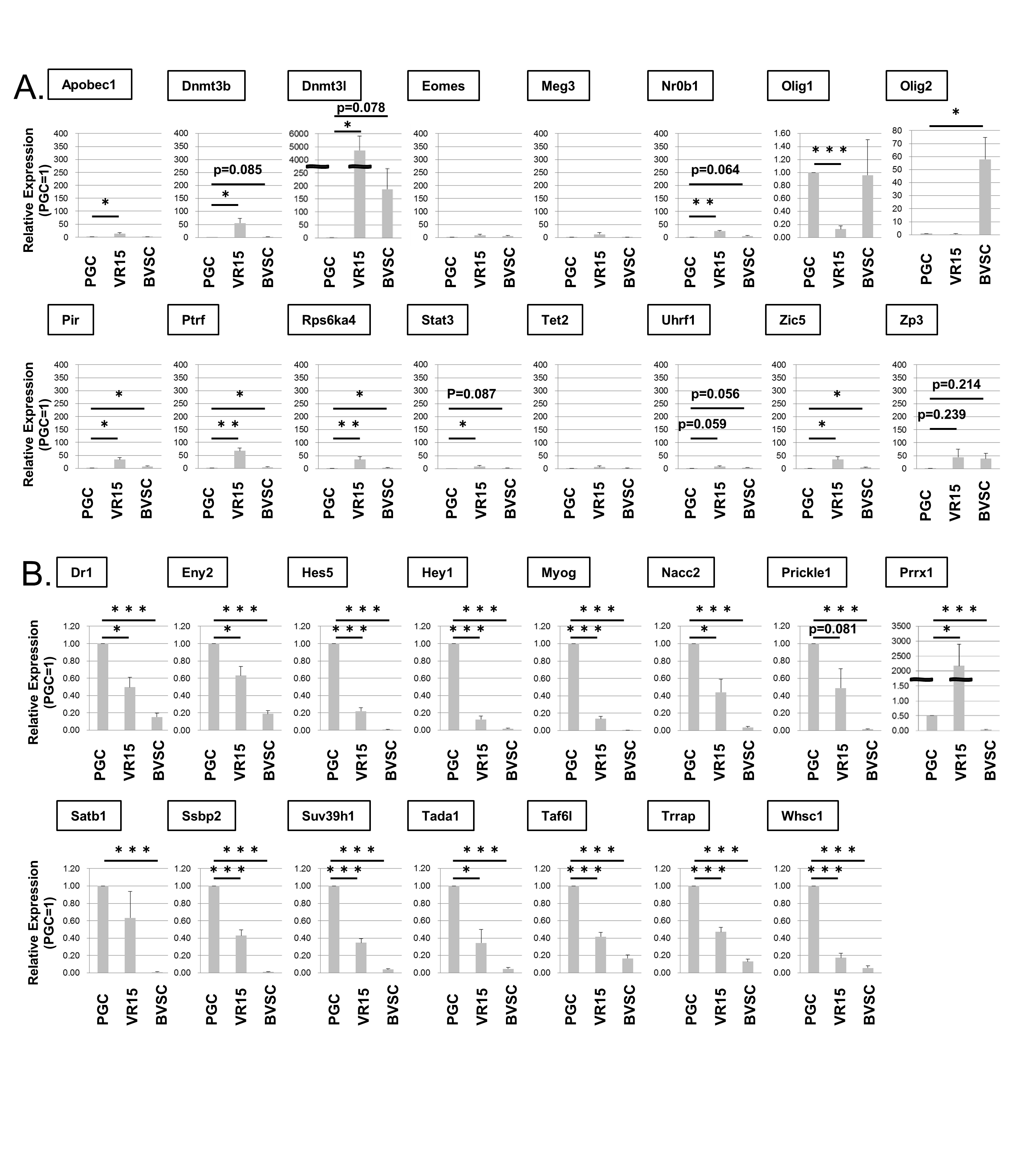

Supplement: S1 Fig — The expression of candidate genes was determined with RT-qPCR, and relative expression levels in VR15-ESCs and BVSC-ESCs compared with those in E12.5 PGCs are shown. Genes whose expression is less than 10 times higher (A) or lower (B) in ESCs compared to PGCs are shown. Error bars show the SE of three independent experiments. *p<0.05, **p<0.01, ***p<0.001. (TIF) [file pone.0205004.s001.tif]

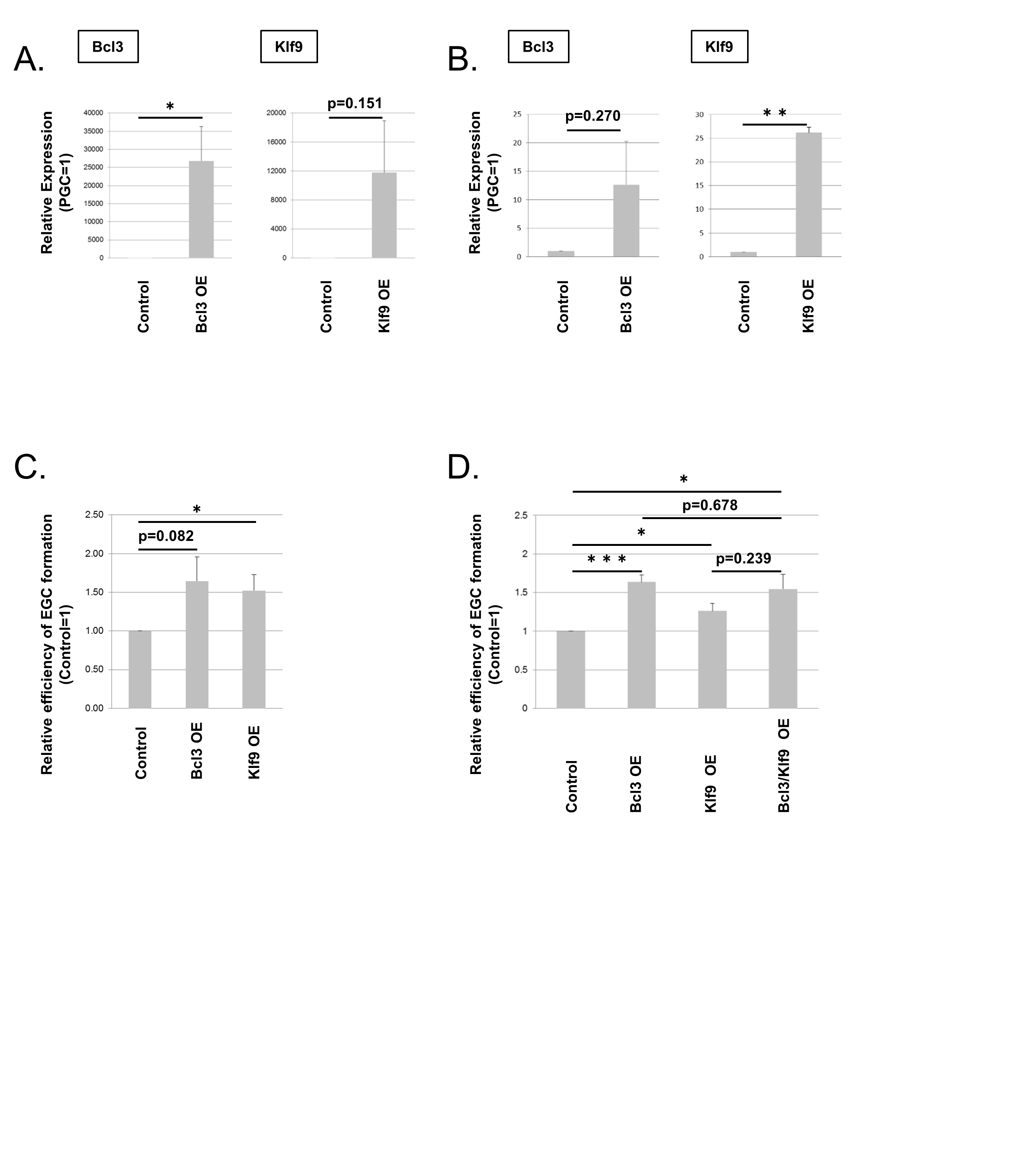

Supplement: S2 Fig — (A, B) Induction of Bcl3 or Klf9 expression in Bcl3-OE or Klf9-OE PGCs by infecting the lenti-virus vectors at MOI 5 (A) and MOI 0.2 (B) after culturing for 2 days. The expression was determined by RT-qPCR. (C, D) Relative efficiency of EGC formation by Bcl3-OE or Klf9-OE (MOI 0.2) (C) or by Bcl3-OE and/or Klf9-OE (MOI 5) (D) in PGCs compared with that of control. EGC colonies are identified by alkaline phosphatase staining. Error bars show the SE of four (A, C, D), two (B) independent experiment. *p < 0.05, **p < 0.01, ***p < 0.001. (TIF) [file pone.0205004.s002.tif]

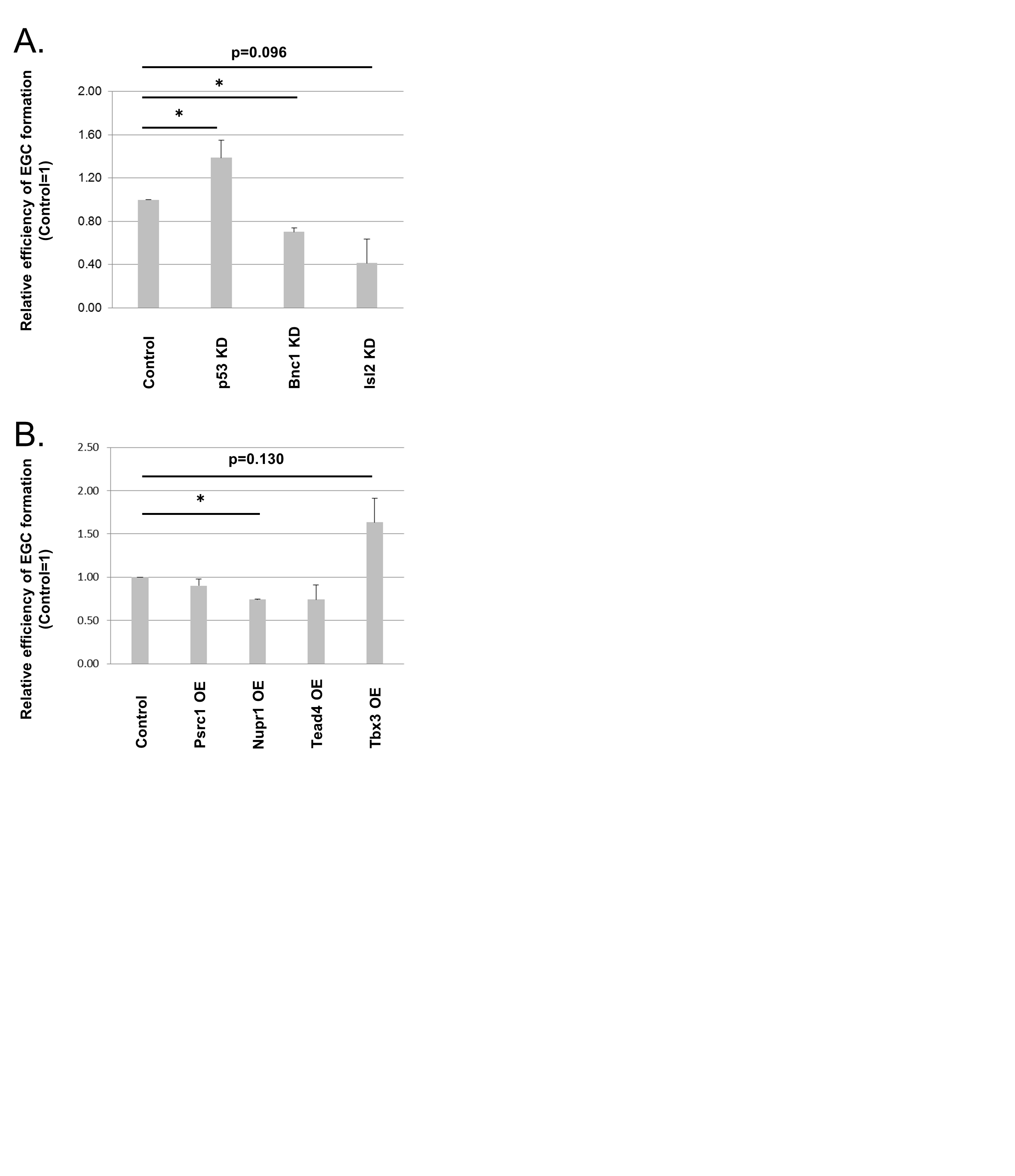

Supplement: S3 Fig — Relative efficiency of EGC formation by Bnc1-KD, Isl2-KD, Nupr1-OE, Psrc1-OE, Tead4-OE, and Tbx3-OE PGCs compared with control is shown. Error bars show the SE of three independent experiment. *p < 0.05. **p < 0.01. (TIF) [file pone.0205004.s003.tif]
